# Pentoxifylline Diminishes the Oxidative Damage to Renal Tissue Induced by Streptozotocin in the Rat

**DOI:** 10.1080/154386090897974

**Published:** 2004

**Authors:** M. E. Dávila-Esqueda, F. Martínez-Morales

**Affiliations:** 1 Department of Pharmacology and Physiology Faculty of Medicine Universidad Autónoma de San Luis Potosí 2405 Carranza Avenue, Los Filtros S.L.P. México San Luis Potosi 78210 Mexico

## Abstract

Oxidative damage has been suggested to be a contributing factor in the development to diabetic nephropathy (DN). Recently, there has been evidence that pentoxifylline (PTX) has free radical-scavenging properties; thus, its antiinflammatory and renoprotective effects may be related to a reduction in reactive oxygen species production. It is likely that the pharmacological effects of PTX include an antioxidant mechanism as shown in in vitro assays. The aim of this study was to evaluate whether the reported renoprotective effects of PTX could be the result of its antioxidant actions in streptozotocin (STZ)-induced DN in rats. The administration of PTX over a period of 8 weeks, in addition to displaying renoprotective effects, caused a significant reduction in lipoperoxide levels (LPOS) in the diabetic kidney (*P* < 0.05), compared to untreated rats. These levels were comparable to those in the healthy kidney of experimental animals (*P* > 0.05). All untreated STZ rats exhibited an increase in LPOS as opposed to healthy controls (H) (*P* < 0.001). The total antioxidant activity (TAA) in plasma was increased significantly already after 2 days of STZ (*P* < 0.05). When we examined the progression of TAA in STZ rats, there was a significant decrease over 8 weeks (*P* < 0.05). PTX treatment caused an increase in TAA when compared to untreated STZ rats (*P* < 0.05). Renal hypertrophy was less evident in PTX-treated STZ than in untreated STZ rats, evaluated by kidney weight/body weight ratio. These results indicate that PTX decreases the oxidative damage induced by these experimental procedures and may increase antioxidant defense mechanisms in STZ-induced diabetes in rats.


*Experimental Diab. Res.*, 5:245–251, 2004
Copyright © Taylor and Francis Inc.
ISSN: 1543-8600 print / 1543-8619 online
DOI: 10.1080/154386090897974

Taylor & Francis
Taylor & Francis Group

### Pentoxifylline Diminishes the Oxidative Damage to Renal Tissue Induced by Streptozotocin in the Rat

**M. E. Dávila-Esqueda and F. Martínez-Morales**

*Department of Pharmacology and Physiology, Faculty of Medicine, Universidad Autónoma de San Luis Potosí, San Luis Potosi, México*

**Oxidative damage has been suggested to be a contributing factor in the development to diabetic nephropathy (DN). Recently, there has been evidence that pentoxifylline (PTX) has free radical-scavenging properties; thus, its anti-inflammatory and renoprotective effects may be related to a reduction in reactive oxygen species production. It is likely that the pharmacological effects of PTX include an antioxidant mechanism as shown in in vitro assays. The aim of this study was to evaluate whether the reported renoprotective effects of PTX could be the result of its antioxidant actions in streptozotocin (STZ)-induced DN in rats. The administration of PTX over a period of 8 weeks, in addition to displaying renoprotective effects, caused a significant reduction in lipoperoxide levels (LPOS) in the diabetic kidney ($P < 0.05$), compared to untreated rats. These levels were comparable to those in the healthy kidney of experimental animals ($P > 0.05$). All untreated STZ rats exhibited an increase in LPOS as opposed to healthy controls (H) ($P < 0.001$). The total antioxidant activity (TAA) in plasma was increased significantly already after 2 days of STZ ($P < 0.05$). When we examined the progression of TAA in STZ rats, there was a significant decrease over 8 weeks ($P < 0.05$). PTX treatment caused an increase in TAA when compared to untreated STZ rats ($P < 0.05$). Renal hypertrophy was less evident in PTX-treated STZ than in untreated STZ rats, evaluated by kidney weight/body weight ratio. These results indicate that PTX decreases the oxidative damage induced by these experimental procedures and may increase antioxidant defense mechanisms in STZ-induced diabetes in rats.**

**Keywords** Oxidative Damage; Diabetic Nephropathy; Antioxidants; Reactive Oxygen Species

### INTRODUCTION

Diabetic nephropathy (DN) is one of the main causes of end-stage renal disease. The metabolic events responsible for its development are not yet completely understood. Poor glycemic control undoubtedly plays a significant part, as shown by both clinical [1] and pathological studies [2]. Among all mechanisms known to trigger and worsen DN, the production of reactive oxygen species (ROS) may play a crucial role. Possible mediators of untoward effects of hyperglycemia include advanced glycation end products (AGEs), known to accumulate in tissues as a function of time and glucose concentration [3]. These harmful products promotes ROS formation in cells and tissues [4, 5]. AGEs and ROS-induced cellular damage promote dysfunctions that interfere with the genetic expression of peptides and cytokines. Hence perturbed redox balance may determine cell proliferation and vascular function during the development of diabetic complications [6–8]. Thus, these responses may play a crucial role in the genesis of DN.

Evidence suggests that in the early stages of diabetes, appropriate interventions aimed at improving glycemic control, lowering hypertension and decreasing microalbuminuria [1, 9] can significantly delay the progression of renal disease. Drugs used in the treatment of other diseases have shown pharmacological properties that preserve or even ameliorate renal function. Angiotensin-converting enzyme inhibitors (ACEI) have been used with relatively good success in treating DN [10]. However,

Received 6 June 2004; accepted 5 October 2004.
M. E. D. received a fellowship (92290) from CONACyT-Mexico during her doctoral training at UASLP. This work was supported by grants 19980202034 from SIHGO-CONACyT and 25887-M from the National Council of Sciences (CONACyT-Mexico) presented to F. M.
Address correspondence to M. E. Dávila Esqueda, Department of Pharmacology, Faculty of Medicine, UASLP, 2405 Carranza Avenue, 78210, Los Filtros S.L.P. México. E-mail: medavila@uaslp.mx pharmacological alternatives must be explored, since some patients with DN cannot be treated with ACEI due to intolerance or allergic reactions to these drugs. In this context, PTX, a methylxanthine derivative with rheological properties, has been proposed as a protector of renal function in DN. Several studies have reported that PTX reduces urinary protein excretion in patients with type 2 diabetes progressing toward renal dysfunction and advanced renal failure [11–13], as well as in experimental nephritic syndrome [14]. Methylxanthines and their derivates are nonspecific phosphodiesterases inhibitors (PDEI) most frequently used for the modulation of macrophage functions [15]. They are recognized as powerful suppressors of inflammatory cell function. However, the precise mechanism of their action is still poorly understood.

Recently, PTX has gained considerable interest as a ROS scavenger. Several in vitro studies have confirmed the potential antioxidant effects of this drug [16–19]. Although PTX has been established as a renoprotective drug, its contribution in protecting the kidney from high glucose toxicity by damping ROS production has not been investigated in DN where oxidative stress is involved. Thus, the purpose of this study was to investigate the effect of PTX administration on lipoperoxides (LPOS = malondialdehyde and 4-hydroxy-2 nonenal, as an index of lipid peroxidation in the kidney) and total antioxidant activity (TAA) and to correlate the results with renoprotective effect in STZ-induced diabetes in rats. We also examined renal and biochemical parameters at 2 days after STZ and after the 4th and 8th weeks of PTX treatment.

### MATERIALS AND METHODS

### Animals

Studies were performed using male Sprague-Dawley rats weighing 230 to 290 g. The guidelines of our institution for the care and use of laboratory animals was observed. The rats were fed with standard diet and were given water ad libitum.

#### *Diabetes Induction*

Diabetes was induced by a single intraperitoneal (i.p) injection of streptozotocin (65 mg/kg body weight [b.w.]) freshly dissolved in sterile saline. Healthy control rats (H) received equivalent amounts of saline i.p. Blood samples were drawn 2 days later and the diabetes was confirmed by measuring the level of blood glucose, using a reflectance meter (One Touch Basic, LifeScan J & J, Milpitas, CA, USA). Only rats with blood glucose levels $\geq$ 250 mg/dL were included. Three groups ($n = 9$ each) were established by a randomized method as follows: control group (H), diabetic group without treatment (STZ), and pentoxifylline-treated diabetic group (STZ + PTX). The latter group received daily doses of PTX at 80 mg/kg b.w. administered i.p. whereas the H and STZ group received only placebo. The animals used were housed in metabolic cages, maintained at 24°C and constant humidity (60%) with a 12 h light:dark cycle. Urine volume and water and food intake per 24 hrs were monitored daily during the whole experiment. Furthermore at day 2, and then every week body weight of the animals was monitored.

Animals were sacrificed after 4- and 8-weeks of diabetes. Rats were weighed prior to sacrifice, and 24-h urine samples were collected to determine albumin excretion rate ($U_{alb}V$). Additionally, blood samples were drawn to quantify TAA, and the kidneys were removed, weighed and analyzed for LPOS (malondialdehyde and 4-hydroxy-2-nonenal) as an index of lipid peroxidation.

#### *Tissue Preparation*

Rats were anesthetized with ether. Blood samples were obtained by cardiac puncture and collected in EDTA tubes in order to analyze the following biochemical parameters; creatinine, glucose, glycosylated hemoglobin fraction % $HbA_{1c}$, total cholesterol, and triglycerides. Kidneys were removed, cleaned of perirenal tissue, blotted dry and weighed. Subsequently, kidneys were processed for LPOS determination. Minced renal tissue was homogenized in 20 mM phosphate buffer (pH 7.4) containing butylated hydroxytoluene (BTH) at a final concentration of 5 mM at 4°C for 30 sec (2 × 15 sec with a 15-sec cooling interval) using a polytron homogenizer (Teckman Model TR-10, Cincinnati, OH, USA). The homogenate was centrifuged at 3000 × g for 10 min at 4°C (Sorvall RC-5B, Du pont Instruments, Wilmington, DE, USA ). The resultant supernatant was used to measure LPOS by the colorimetric assay BIOXYTECH, LPO 586 (Oxis International, Inc., Portland, OR USA).

#### *Biochemical Assays*

$HbA_{1C}$ was measured using the tina-quant assay [20] in an automatic analyzer. Total plasma concentrations of cholesterol, triglycerides and creatinine were measured enzymatically in a Hitachi 911 clinical chemistry analyzer (Manufacturer Roche/BMC, Palo Alto, CA USA). TAA was measured by a colorimetric technique as described by Miller et al. [21] using a commercial kit (Randox Laboratories LTD, UK). Twenty-four-hour urine samples were obtained from animals kept in metabolic cages with access only to drinking water. Creatinine was measured as well as albumin concentration determined by radioimmunoassays (Euro/Diagnostic Products Corporation, San Luis Potosi, LA, USA).

*Preparation of Pancreas Sections and Histochemical Analyses*

The rats were anesthetized using pentobarbital sodium. A midline abdominal incision was made and pancreas was removed from the rat and fixed overnight in a solution of 10% Formaldehyde. Fixed tissues were processed routinely for paraffin embedding and ~5 $\mu$m sections were prepared and mounted on slides. Staining was performance on this slides with Hematoxylin and Eosin.

### Statistical Data Analysis

Results were expressed as means ± standard error of the mean (SEM). The results obtained for the quantitative factors studied (glucose, %$HbA_{1C}$, total plasma cholesterol and triglycerides, $U_{alb}V$ and 24-h creatinine clearance: $C_{Cr}$) were compared among the groups by applying one-way analysis of variance test. Significance was set at $P < 0.05$ in all cases.

### RESULTS

All STZ rats exhibited biochemical signs of diabetes. They presented hyperglycemia, with blood concentrations higher than 250 mg/dL 2 days after STZ administration (Table 1), thus confirming the diabetic state of the animals. Additionally, hypercholesterolemia and hypertriglyceridemia were observed at 2 days of diabetes and remained elevated throughout the experiment.

Levels of blood glucose and plasma total cholesterol and triglycerides did not differ between untreated rats and PTX-treated STZ rats at the end of treatment. Mean blood glucose levels, as measured by $HbA_{1C}$, were consistently higher in STZ rats at the fourth week, reflecting and verifying the hyperglycemic condition during the experiment. A progressive decrease in $HbA_{1C}$ was seen in PTX-treated STZ rats, reaching statistical significance after 8 weeks of treatment (7.21 ± 0.40 vs. 9.09 ± 0.40% untreated STZ rats, $P < 0.05$). Conversely, there was a progressive increase in untreated STZ rats (9.09 ± 0.40 vs. 4.86 ± 0.99%, $P < 0.05$).

Table 2 shows changes in renal functional parameters. At the beginning of the experiment, urinary volume (UV) was significantly increased in STZ rats in comparison to healthy rats (83.25 ± 3.62 vs. 21.80 ± 1.31 mL/24 h, $P < 0.0001$), a functional hallmark of the diabetic state. Significantly increased urine volumes persisted in 4- and 8- week untreated STZ and PTX-treated STZ rats.

As expected, uncontrolled diabetes resulted in glomerular hyperfiltration (1.09 ± 0.23 mL/min/100 g b.w., $P < 0.05$ at 4 weeks), remaining high at the end of the experiment, this high rate was partially reversed by PTX therapy (to 0.77 ± 0.06 mL/min/100 g b.w., $P > 0.05$).

There was a significant increase in $U_{alb}V$ at 2 days of diabetes (0.40 ± 0.07 vs. 0.0020 ±0.0001 mg/day before STZ, $P < 0.0001$), a clear progression toward proteinuria in the untreated STZ rats (0.70 ± 0.24 mg/day by week 8) was observed. On the other hand, the PTX-treated STZ rats showed a prevention at increased $U_{alb}V$ (0.27 ± 0.15 mg/day, $P < 0.05$) at the end of the experiment.

LPOS levels were significantly elevated in all STZ rats at 2 days after STZ compared to those in the healthy control group,

**TABLE 1**

Effects of streptozotocin-induced diabetes and pentoxifylline on metabolic parameters measured at 2 days and at 4 and 8 weeks

| Animal group | Glucose (mg%) | %$HbA_{1C}$ | Cholesterol (mg%) | Triglycerides (mg%) |
|---|---|---|---|---|
| H (n = 9) | | | | |
| 2 days | 95.2 ± 9.00 | 4.95 ± 0.63 | 46.90 ± 3.23 | 63 ± 0.13 |
| 4 weeks | 94.8 ± 10.70 | 5.37 ± 0.24 | 44.50 ± 3.16 | 84 ± 12.55 |
| 8 weeks | 102.6 ± 15.30 | 4.85 ± 0.07 | 45.50 ± 5.20 | 64 ± 7.93 |
| STZ (n = 9) | | | | |
| 2 days | 314.5 ± 14.60* | 4.86 ± 0.99 | 98.85 ± 8.89* | 283 ± 45.02* |
| 4 weeks | 358.3 ± 48.90* | 8.37 ± 0.27* | 87.91 ± 12.63* | 250 ± 69.41* |
| 8 weeks | 282.4 ± 21.70* | 9.09 ± 0.40*[,b] | 88.78 ± 14.57* | 334 ± 178.46* |
| STZ + PTX (n = 9) | | | | |
| 2 days | 273.2 ± 14.20* | 4.62 ± 1.40 | 72.18 ± 7.41* | 367 ± 83.81* |
| 4 weeks | 289.5 ± 23.70* | 8.32 ± 0.05* | 76.82 ± 8.67* | 152 ± 42.69* |
| 8 weeks | 296.7 ± 12.50* | 7.21 ± 0.40*[,a] | 71.86 ± 18.77* | 244 ± 75.33* |

Data are means ± SEM, *$P < 0.05$ significantly different from the healthy rats.
[a]Significantly different from STZ + PTX group at 2 days, $P < 0.05$.
[b]Significantly different from STZ + PTX group at 8 weeks, $P < 0.05$.
n = number of animals.

**TABLE 2**
Renal function in STZ and STZ + PTX rats

| Animal group | UV (mL/24 h) | CCr (mL/min/100 g b.w.) | $U_{alb}V$ (mg/day) |
|---|---|---|---|
| H (n = 9) | | | |
| 2 days | 21.80 ± 1.31 | 0.85 ± 0.10 | 0.0020 ± 0.0001 |
| 4 weeks | 20.39 ± 2.18 | 0.94 ± 0.12 | 0.0023 ± 0.0003 |
| 8 weeks | 21.49 ± 3.26 | 1.08 ± 0.12 | 0.0021 ± 0.0003 |
| STZ (n = 9) | | | |
| 2 days | 83.25 ± 3.62* | 0.71 ± 0.08 | 0.40 ± 0.07** |
| 4 weeks | 120.54 ± 14.05* | 1.09 ± 0.23[a] | 0.49 ± 0.15** |
| 8 weeks | 103.20 ± 11.57* | 0.85 ± 0.09 | 0.70 ± 0.24** |
| STZ + PTX (n = 9) | | | |
| 2 days | 79.92 ± 5.58* | 0.98 ± 0.15 | 0.40 ± 0.15** |
| 4 weeks | 100.67 ± 9.18* | 0.77 ± 0.06 | 0.46 ± 0.72** |
| 8 weeks | 98.91 ± 7.28* | 0.82 ± 0.08 | 0.27 ± 0.15**,[b] |

Data are means ± SEM, *$P < 0.05$ significantly different from H group at 2 days and 4 and 8 weeks.
**$P < 0.0001$ significantly different from H group at 2 days and 4 and 8 weeks.
[a]Significantly different from STZ rats at 2 days; $P < 0.05$.
[b]Significantly different from STZ rats at 8 weeks; $P < 0.05$.
UV = urinary volume, CCr = creatinine clearance, $U_{alb}V$ = albumin excretion rate, n = number of animals.

as shown in Table 3 (4.5 ± 0.7 and 5.3 ± 0.5 vs. 2.0 ± 0.3 $\mu$M LPO/g, both $P < 0.05$). LPOS levels increased during the 8 weeks in untreated STZ-rats (4.5 ± 0.7 vs. 9.9 ± 1.7 $\mu$M LPO/g, $P < 0.05$). On the other hand PTX-treatment was associate with a decline in LPOS ( 5.3 ± 0.5 vs. 3.5 ± 1.1 $\mu$M LPO/g, $P < 0.05$).

**TABLE 3**
Effect of STZ-induced diabetes and PTX on LPOS and TAA measured at t = 0 (2 days), 4 weeks and 8 weeks

| Animal group | $\mu$M LPOS/g tissue | TAA mmol/L |
|---|---|---|
| H (n = 9) | | |
| 2 days | 2.0 ± 0.3 | 1.0 ± 0.3 |
| 4 weeks | 2.3 ± 0.6 | 1.2 ± 0.3 |
| 8 weeks | 2.1 ± 0.4 | 0.9 ± 0.2 |
| STZ (n = 9) | | |
| 2 days | 4.5 ± 0.7* | 2.4 ± 0.5* |
| 4 weeks | 6.4 ± 0.8* | 3.4 ± 0.9* |
| 8 weeks | 9.9 ± 1.7**,[a] | 1.6 ± 0.2[b] |
| STZ + PTX (n = 9) | | |
| 2 days | 5.3 ± 0.5* | 2.9 ± 0.5* |
| 4 weeks | 4.5 ± 0.4* | 1.8 ± 0.4* |
| 8 weeks | 3.5 ± 1.1* | 3.8 ± 0.2[c] |

Data are means ± SEM,
*$P < 0.05$ significantly different from H rats at 2 days.
**$P < 0.001$ significantly different from H rats at 8 weeks.
[a]$P < 0.05$ significantly different from STZ + PTX rats at 8 weeks.
[b]$P < 0.05$ significantly different from STZ rats at 2 days.
[c]$P < 0.05$ significantly different from STZ rats at 8 weeks.
LPOS = lipoperoxides, TAA = total antioxidant activity.

LPOS levels in the PTX-treated STZ rats at 8 weeks did not differ from those of the healthy group (3.5 ± 1.1 vs. 2.1 ± 0.41 $\mu$M LPO/g, $P > 0.5$). TAA in plasma at the beginning of the experiment (2 days) was found to be significantly increased in STZ rats compared to healthy rats (2.40 ± 0.5 vs. 1.0 ± 0.26 mmol/L, $P < 0.05$). When we examined the progression of TAA in STZ rats, this activity decreased significantly over the 8 week period (2.4 ± 0.5 vs. 1.6 ± 0.2 mmol/L, $P < 0.05$). PTX-treated STZ rats at the end of the experiment had increased TAA levels when compared to untreated STZ rats (3.8 ± 0.2 vs. 1.6 ± 0.2 mmol/L, $P < 0.05$).

During the experiment body weight diminished in rats with untreated STZ diabetes compared to healthy rats (266.6 ± 14.12 vs. 355.7 ± 8.77 g, $P < 0.05$; Table 4). The food intake was equally increased in STZ and PTX-treated STZ-rats at 4 and 8 weeks.

Kidney weight/body weight ratio, an index of renal hypertrophy (Table 5), was increased in both STZ and PTX-treated STZ-rats, although this tended to be less in PTX-treated STZ rats.

The diabetogenic action of STZ was quantitatively evaluated by counting the pancreatic $\beta$ cells number with a light microscope, 2 days after its administration, also at 8 weeks of the experiment, finding a severe reduction in this parameter, in comparison to healthy rat (2 ± 2 vs. 10 ± 2 $\beta$ cells number/10 fields, $P < 0.05$, at 8 weeks). An increase of these cells, was

**TABLE 4**
Effect of STZ-induced diabetes and PTX on body weight and food intake/24h measured at t = 0 (2 days), 4 weeks and 8 weeks

| Animal group | Body weight (g) | Food intake (g/24 h) |
|---|---|---|
| H (n = 9) | | |
| 2 days | 260.00 ± 3.86 | 23.80 ± 1.70 |
| 4 weeks | 333.22 ± 14.30[a] | 26.01 ± 2.02 |
| 8 weeks | 355.70 ± 8.77[a] | 27.32 ± 1.33 |
| STZ (n = 9) | | |
| 2 days | 284.29 ± 10.84 | 22.32 ± 1.33 |
| 4 weeks | 261.22 ± 11.52* | 33.66 ± 1.64* |
| 8 weeks | 266.60 ± 14.12** | 32.72 ± 3.03** |
| STZ + PTX (n = 9) | | |
| 2 days | 278.36 ± 7.18 | 21.79 ± 1.50 |
| 4 weeks | 242.43 ± 18.55* | 35.50 ± 1.22* |
| 8 weeks | 277.91 ± 23.21** | 35.29 ± 1.75** |

Data are means ± SEM,
*$P < 0.05$ significantly different from H rats at 4 weeks.
**$P < 0.05$ significantly different from H rats at 8 weeks.
[a]$P < 0.000001$ significantly different from H rats at 2 days.

observed in the PTX-treated STZ-rats at the end of the experiment. (2 ± 2 vs. 6 ± 1 $\beta$ cells number/10 fields, $P < 0.05$).

### DISCUSSION

Our results provide evidence that the administration of PTX to STZ rats over a period of 8 weeks, in addition to displaying renoprotective effects, causes a highly significant reduction in LPOS levels in the diabetic kidney to levels comparable to those in the kidney of non-diabetic animals. The antioxidant system as reflected by TAA was increased after 8 weeks of treatment, suggesting that the PTX could scavenge ROS and potentiate the activity of the antioxidant system in this model.

It has been suggested that antioxidant treatment preserves pancreatic $\beta$ cell function through reduction of oxidative stress, as well as by diminishing $\beta$ cells apoptosis without influencing the rate of $\beta$ cell proliferation [22]. This observation is compatible with our experimental observation of $\beta$ cells number at the end of the PTX treatment. This parameter was found to be increased, therefore it seems reasonable that the preservation $\beta$ cells through improved glycemic control by PTX. In this study we evaluated $HbA_{1c}$ as an index of metabolic control. This $HbA_{1c}$ was decreased at the end of the study in the treated rats. These findings are in concordance with previous evidence reporting that PTX could inhibit glycation [23] reducing glucose auto- oxidation, diminishing the oxidative damage to pancreatic $\beta$ cell, with improvement in insulin secretion.

Reactive oxygen species (ROS) have been invoked as important signal mediators for cell surface receptors, acting as signaling agents in the regulation of several cellular processes, including those during the cell cycle entry [24–26]. Likewise Chen et al. [27] have reported that PTX suppresses the activation and proliferation of mesangial cells, thereby preventing the development of proteinuria in experimental mesangial proliferative glomerulonephritis, also observed in other experimental models of nephropathy in which renal function was evaluated [14]. Thus, the final antiproteinuric effect observed in the present study and in others may be explained in terms of the free radical scavenging properties of PTX.

Among nonspecific PDEI, the protective effect of PTX in autoimmune disease is well documented [28, 29]. While the beneficial effects of PTX in autoimmunity are usually linked to inhibition of TNF-$\alpha$ production and T-cell proliferation [30]. Recent data have implied the involvement of inducible nitric oxide synthase (iNOS) and nitric oxide (NO) as a mechanism in the protection exerted by PTX in autoimmune diabetes [31]. Moreover, if we take into account its antioxidant properties, these may lead to modulations of iNOS resulting in a down-regulation of NO production, with cytoprotective effects. Therefore, an antioxidant mechanism may be responsible for its beneficial actions.

Our data indicate that renal hypertrophy was prevented by PTX treatment, thereby normalizing renal function. Changes in renal function in untreated STZ rats were distinguished by glomerular hyperfiltration and microalbuminuria, and established renal hypertrophy.

Because diabetes is considered a chronic illness and its complications may occur earlier than expected, it was necessary to determine whether STZ-induced changes that occurred during the time of the experiment could be prevented by PTX treatment after 4 to 8 weeks. In this study, we observed that 8 weeks of PTX treatment in experimental diabetes reduced LPOS and increased TAA. The effect was not evident until after 4 weeks treatment. The decrease in TAA in untreated STZ rats after 8 weeks may be the consequence of a impaired antioxidative

**TABLE 5**
Kidney weight/body weight ratio and pancreatic $\beta$ cells number

| Animal group n = 9 | Kidney weight/body weight ratio g/100 g | Pancreatic $\beta$ cells number/10 fields |
|---|---|---|
| H | 0.397 ± 0.008 | 10 ± 2 |
| STZ | 0.576 ± 0.028* | 2 ± 2* |
| STZ + PTX | 0.512 ± 0.020* | 6 ± 1*[,a] |

Data are means ± SEM,
*$P < 0.05$ significantly different from H rats.
[a]$P < 0.05$ significantly different from STZ rats.

mechanisms through glycation of scavenging enzymes. Likewise, gene expression of antioxidant enzymes in pancreatic islets has been shown to be decreased by Lenzen et al. [32].

The usefulness of antioxidants in the treatment of diabetes has been reported in several studies, mainly in preventing complications and maintaining plasma glucose levels. Administration of antioxidants has been shown to improve insulin efficiency and glycemic equilibrium. Interestingly, these drugs potentiate the antihypertensive action of insulin in rats [33].

Some antidiabetic agents may have antioxidant properties independent of their role in glucose control. It also has been shown that thiazolidinediones and sulfonylureas [34] possess these properties. Therefore, in addition to their effectiveness in glycemic control, these drugs have an antioxidant activity that has not been evaluated in DN.

In summary, the present results suggest that multiple mechanisms are likely to mediate cellular injury in response to oxidative stress generated during the development of complications of diabetes and that they are potentially subject to modulation by antioxidant therapy. Thus, this study supports the hypothesis that the spectrum of pharmacological effects exerted by PTX may be attributed to its ability to scavenge ROS. Therefore, treatment with PTX may offer metabolic benefits resulting in a decrease in the progression of renal disease in experimental diabetes.

### REFERENCES

[1] The Diabetes Control and Complications Trial (DCCT) Research Group. (1995) Effect of intensive therapy on the development and progression of diabetic nephropathy in the Diabetes Control and Complications Trial. *Kidney Int.,* **47,** 1703–1720.

[2] Bangstad, H. J., Osterby, R., Dahl-Jorgensen, K., Berg, K. J., Hartmann, A., and Hanssen, K. F. (1994) Improvement of blood glucose control in IDDM patients retards the progression of morphological changes in early diabetic nephropathy. *Diabetologia,* **37,** 483–490.

[3] Dyer, D. G., Dunn, J. A., Thorpe, S. R., Bailie, K. E., Lyons, T. J., McCance, D. R., and Baynes, J. W. (1993) Accumulation of Maillard reaction products in skin collagen in diabetes and aging. *J. Clin. Invest.,* **91,** 2463–2469.

[4] Brownlee, M., Cerami, A., and Vlassara, H. (1988) Advanced glycosylation end products in the tissue and the biochemical basis of diabetic complications. *N. Engl. J. Med.,* **318,** 1315–1321.

[5] Sakurai, T., and Tsuchiya, S. (1988) Superoxide production from nonenzymatically glycation protein. *FEBS Lett.,* **236,** 406–410.

[6] Gohil, K., Roy, S., Packer, L., and Sen, C. K. (1999) Antioxidant regulation of gene expression: analysis of differentially expressed mRNAs. *Methods Enzymol.,* **300,** 402–410.

[7] Senk, C. K., and Packer, L. (1996) Antioxidant and redox regulation of gene transcription. *FASEB J.,* **10**(7), 109–120.

[8] Schmidt, A. M., Hori, O., Cao, R., Yan, S. D., Brett, J., Wautier, J. L., Ogawa, S., Kuwabara, K., Matsumoto, M., and Stern, D. (1986) RAGE: A novel cellular receptor for advanced glycation end products. *Diabetes,* **45**(Suppl 3), S77–S80.

[9] Mogensen, C. E., Keane, W. F., Bennett, P. H., Jerumus, G., Parving, H. H., Pasa, P., Steffess, M. W., Striker, G. E., and Viberti, G. C. (1995) Prevention of diabetic renal disease with special reference to microalbuminuria. *Lancet,* **346,** 1080–1084.

[10] Viberti, G., Mogensen, C. E., Groop, L. C., and Pauls, J. F. (1994) Effect of Captopril on progression to clinical proteinuria in patients with insulin-dependent diabetes mellitus and microalbuminuria. *JAMA,* **271**(4), 275–279.

[11] Navarro, J. F., and Mora, C. (1999) Antiproteinuric effect of pentoxifylline in patients with diabetic nephropathy. *Diabetes Care,* **22**(6), 1006–1008.

[12] Guerrero-Romero, F., Rodríguez-Moran, M., Paniagua-Sierra, J. R., Garcia-Bulnes, G., Salas-Ramírez, M., and Amato, D. (1995) Pentoxifylline reduces proteinuria in insulin-dependent and non-insulin-dependent diabetic patients. *Clin. Nephrol.,* **43**(2), 116–121.

[13] Tripathi, K., Prakash, J., Appaiha, D., and Srivastava, P. K. (1993) Pentoxifylline in management of proteinuria in diabetic nephropathy. *Nephron.,* **64**(4), 641–642.

[14] Berens, K. L., Verani, R. R., and Luke, D. R. (1998) Role of neutrophils and macrophages in experimental nephrosis of the rat. *Renal Failure,* **20**(1), 53–63.

[15] Dominguez-Jimenez, C., Sancho, D., Nieto, M., Montoya, M. C., Barreiro, O., Sanchez-Madrid, F., and Gonzalez-Amaro, R. (2002) Effect of pentoxifylline on polarization and migration of human leukocytes. *J. Leukoc. Biol.,* **71**(4), 588–596.

[16] Horvath, B., Marton, Z., Halmosi, R., Alexy, T., Szapary, L., Vekasi, J., Biro, Z., Habon, T., Kesmarky, G., and Toth, K. (2002) In vitro antioxidant properties of pentoxifylline, piracetam, and vinpocetine. *Clin. Neuropharmacol.,* **25**(1), 37–42.

[17] Bhat, V. B., and Madyastha, K. M. (2001) Antioxidant and radical scavenging properties of 8-oxo derivatives of xanthine drugs pentoxifylline and lisofylline. *Biochem. Biophys. Res. Commun.,* **288**(5), 1212–1217.

[18] Freitas, J. P., Filipe, P., and Guerra-Rodrigo, F. (1995) Potential antioxidative effects of pentoxifylline. *CR Seances Soc. Biol. Fil.,* **189**(3), 401–405.

[19] Freitas, J. P., and Filipe, P. M. (1995) Pentoxifylline: A hydroxyl radical scavenger. *Biol. Trace Elem. Res.,* **47**(1–3), 307–311.

[20] Jarausch, J., Lotz, J., and Hafner, G. (1996). Reference values for tina-quant (% HbA1C assay. *Clin. Chem.,* **42,** 116.

[21] Miller, N. J., Rice-Evans, C., Davies, M. J., Gopinathan, U., and Milner, A. (1993) A novel method for measuring antioxidant capacity and its application to monitoring the antioxidant status in premature neonates. *Clin. Sci.,* **84,** 407–412.

[22] Kaneto, H., Kajimoto, Y., Miyagawa, J., Matsuoka, T., Fujitani, Y., Umayahara, Y., Hanafusa, T., Matsuzawa, Y., Yamasaki, Y., and Hori, M. (1999) Beneficial effects of antioxidants in Diabetes. Possible protection of pancreatic $\beta$-cells against glucose toxicity. *Diabetes.,* **48**.

[23] Rahbar, S., Natarajan, R., Yerneni, K., Scott, S., Gonzalez, N., and Nadler, J. L. (2000) Evidence that pioglitazide, metformin and pentoxifylline are inhibitors of glycation. *Clin. Chim. Acta,* **301**(1–2), 65–67.

[24] Goldstone, S., and Hunt, N. (1997) Redox regulation of the mitogen activated protein kinase pathway during lymphocyte activation. *Biochim. Biophys. Acta,* **1355,** 353–360.

[25] Goldstone, S. D., Milligan, A. D., and Hunt, N. H. (1996) Oxidative signaling and gene expression during lymphocyte activation. *Biochim. Biophys. Acta,* **1314**(1–2), 175–182.

[26] Lee, J. R. (2003) Reactive oxygen species play roles on $\beta$ cell surface receptor CD40-mediated proximal and distal signaling events: effects of an antioxidant, N-acetyl-L-cysteine treatment. *Mol. Cell Biochem.,* **252**(1–2), 1–7.

[27] Chen, Y. M., Chien, C. T., Hu-Tsai, M. I., Wu, K. D., Tsai, C. C., Wu, M. S., and Tsai, T. J. (1999) Pentoxifylline attenuates experimental mesangial proliferative glomerulonephritis. *Kidney Int.,* **56**(3), 932–943.

[28] Segal, R., Dayan, M., Zinger, H., and Mozes, E. (2001) Suppression of experimental systemic lupus erythematosus (SLE) in mice via TNF inhibition by an anti-TNFalpha monoclonal antibody and by Pentoxiphylline. *Lupus,* **10**(1), 23–31.

[29] Liang, L., Beshay, E., and Prud'homme, G. J. (1998) The phosphodiesterase inhibitors pentoxifylline and rolipram prevent diabetes in NOD mice. *Diabetes,* **47**(4), 570–575.

[30] Park, E., Schuller-Levis, G., Park, S. Y., Jia, J. H., and Levis, W. R. (2001) Pentoxifylline downregulates nitric oxide and tumor necrosis factor-alpha induced by mycobacterial lipoarabinomannan in a macrophage cell line. *Int. J. Lepr. Other Mycobact. Dis.,* **69**(3), 225–233.

[31] Markovick, M., Miljkovic, D. J., and Trajkovic, V. (2003) Regulation of inducible nitric oxide synthase by cAMP-elevating phosphodiesterase inhibitors. *Current Drug Targets Inflammation Allerg.,* **22,** 63–79.

[32] Lenzen, S., Drinkgern, J., and Tiedge, M. (1996) Low antioxidant enzyme gene expression in pancreatic islets compared with various other mouse tissues. *Free Radic. Biol. Med.,* **20,** 463–466.

[33] Koo, J. R., Ni, Z., Oviesi, F., and Vaziri, N. D. (2002) Antioxidant therapy potentates antihypertensive action of insulin in diabetic rats. *Clin. Exp. Hypertens.,* **24**(5), 333–344.

[34] Bonnefont-Rousselot, D. (2001) Antioxidant and anti-AGE therapeutics: Evaluation and perspectives. *J. Soc. Biol.,* **195**(4), 391–398.

